# COVID-19-Associated Bronchiectasis and Its Impact on Prognosis

**DOI:** 10.7759/cureus.15051

**Published:** 2021-05-16

**Authors:** Aasir M Suliman, Bassel W Bitar, Amer A Farooqi, Anam M Elarabi, Mohamed R Aboukamar, Ahmed S Abdulhadi

**Affiliations:** 1 Internal Medicine Department, Hamad Medical Corporation, Doha, QAT; 2 Pulmonology Department, Hamad Medical Corporation, Doha, QAT; 3 Infectious Disease Department, Hamad Medical Corporation, Doha, QAT

**Keywords:** covid-19, coronavirus, bronchiectasis, chest ct, pneumonia

## Abstract

Coronavirus disease 2019 (COVID-19), which initially emerged in Wuhan, China, has rapidly swept around the world, causing grave morbidity and mortality. It manifests with several symptoms, on a spectrum from asymptomatic to severe illness and death. Many typical imaging features of this disease are described, such as bilateral multi-lobar ground-glass opacities (GGO) or consolidations with a predominantly peripheral distribution. COVID-19-associated bronchiectasis is an atypical finding, and it is not a commonly described sequel of the disease. Here, we present a previously healthy middle-aged man who developed progressive bronchiectasis evident on serial chest CT scans with superimposed bacterial infection following COVID-19 pneumonia. The patient's complicated hospital course of superimposed bacterial infection in the setting of presumed bronchiectasis secondary to COVID-19 is alleged to have contributed to his prolonged hospital stay, with difficulty in weaning off mechanical ventilation. Clinicians should have high suspicion and awareness of such a debilitating complication, as further follow-up and management might be warranted.

## Introduction

Beginning in December 2019, a series of pneumonia cases were reported in Wuhan City, Hubei Province, China. Further investigations revealed that it was a new type of viral pneumonia caused by severe acute respiratory syndrome coronavirus 2 (SARS-Cov-2), which was termed coronavirus disease 2019 (COVID-19). Symptoms are variable, nonspecific, and include dry cough, fever, fatigue, myalgia, dyspnea, anosmia, and ageusia [1]. The real-time reverse transcription-polymerase chain reaction (rRT-PCR) test is the current gold standard for confirming infection and is performed using nasal or pharyngeal swab specimens.

Computerized tomography of the thorax (CT thorax), as a routine imaging tool for pneumonia diagnosis, is of great importance in the early detection and treatment of patients affected by COVID-19. Chest CT may detect the early parenchymal abnormalities in the absence of positive rRT-PCR at initial presentation [2]. Since chest CT was introduced as a diagnostic tool for COVID-19 pneumonia, many typical features of this disease were described such as bilateral multi-lobar ground-glass opacification (GGO) with a prevalent peripheral or posterior distribution, mainly in the lower lobes; sometimes, consolidative opacities superimposed on GGOs could be found [3]. To our knowledge, bronchiectasis is not a classical finding in COVID-19 pneumonia, with a paucity of reporting on its development and progression during the disease course.

## Case presentation

**Figure 1:**
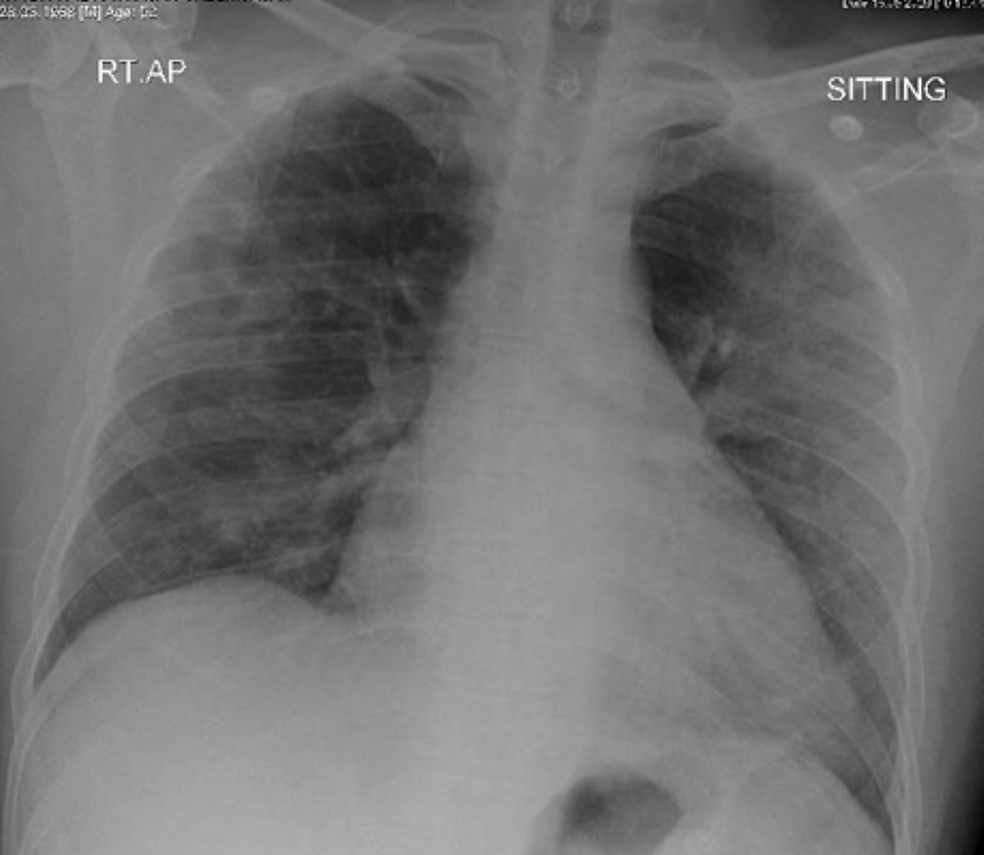
Bilateral predominantly peripheral alveolar infiltrates with loss of left hemidiaphragm silhouette on anteroposterior chest X-ray

A 52-year-old male, with no comorbidities, presented to the emergency department with a 10-day history of fever and dry cough followed by progressive exertional shortness of breath. The patient is a non-smoker and worked as a driver. Examination showed a febrile, ill-looking patient, in respiratory distress, with a respiratory rate of 36 breaths per minute and requiring 6 L/min of Oxygen via a nasal cannula. Chest auscultation was significant for bilateral crackles, without evidence of raised jugular venous pressure (JVP) or lower limb edema. Other examination findings were within normal parameters. The initial investigation was significant for white blood cell (WBC) - 17 x10^3/uL, C-reactive protein (CRP) - 131 mg/L, ferritin - 836 ug/L, and bilateral pulmonary infiltrates in chest X-ray (CXR). (Figure 1). COVID-19 rRt-PCR tested positive; hence was diagnosed with severe COVID‑19 pneumonia and was started on treatment according to the COVID-19 management protocol.

**Figure 2:**
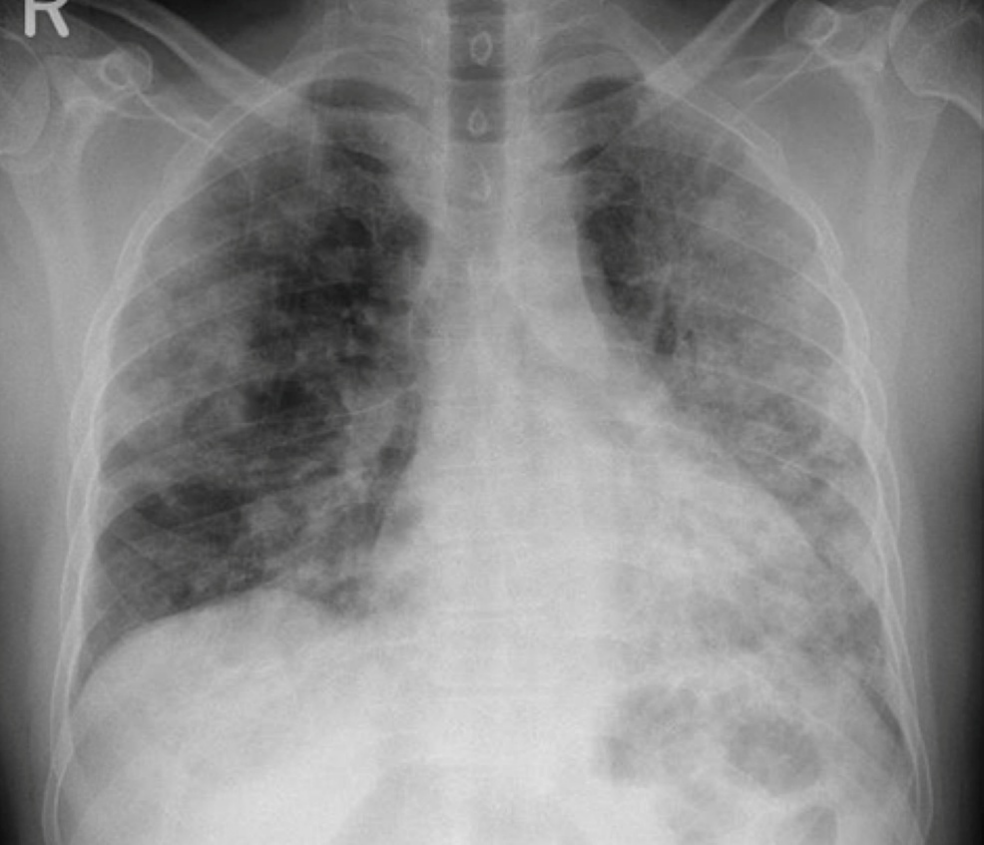
Progression of the bilateral pulmonary consolidation on chest X-ray

Over the subsequent five days, his oxygen requirements gradually increased, reaching 11 L/min via a non-rebreather mask. A follow-up chest x-ray showed progression of previously seen bilateral infiltrates (Figure 2).

**Figure 3:**
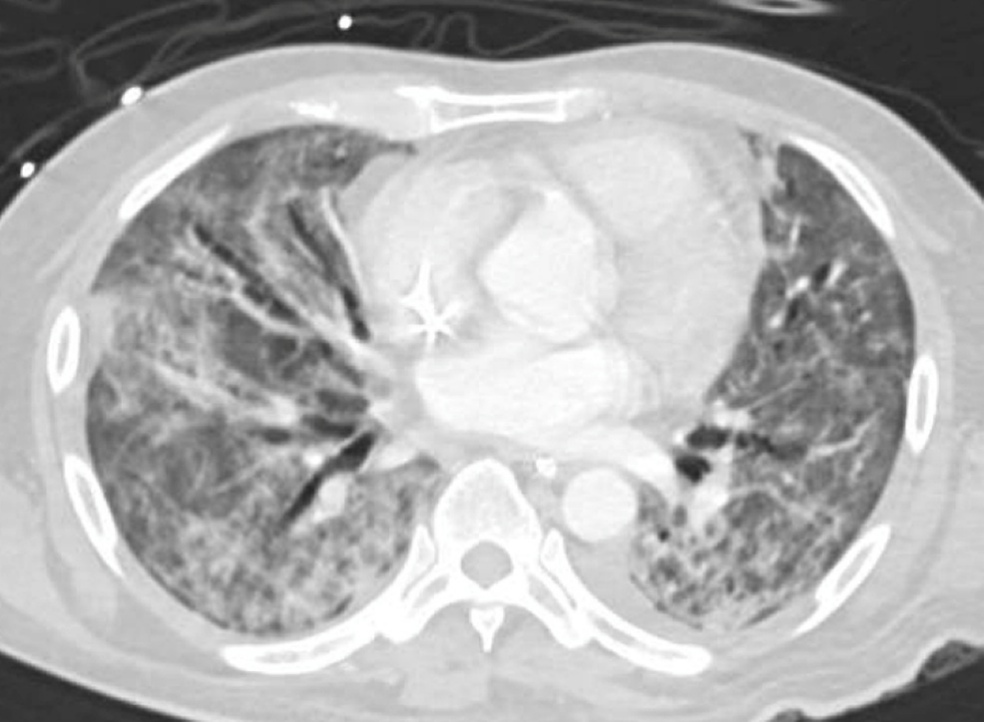
CT-chest showing bilateral consolidation and ground-glass opacities with air bronchogram and evidence of bronchiectasis more on the right side

He was started on methylprednisolone and non-invasive ventilation. Two weeks into admission, his condition continued to deteriorate and endotracheal intubation with mechanical ventilation was required. Two weeks later, the patient started to have a high-grade fever, with further sepsis workup revealing Pseudomonas aeruginosa and Stenotrophomonas maltophilia in tracheal aspirate culture; hence started on piperacillin-tazobactam and teicoplanin. CT thorax was done and revealed bilateral diffuse ground-glass infiltrates and airspace involving almost all lung segments and minimal left-sided pleural effusion (Figure 3).

**Figure 4:**
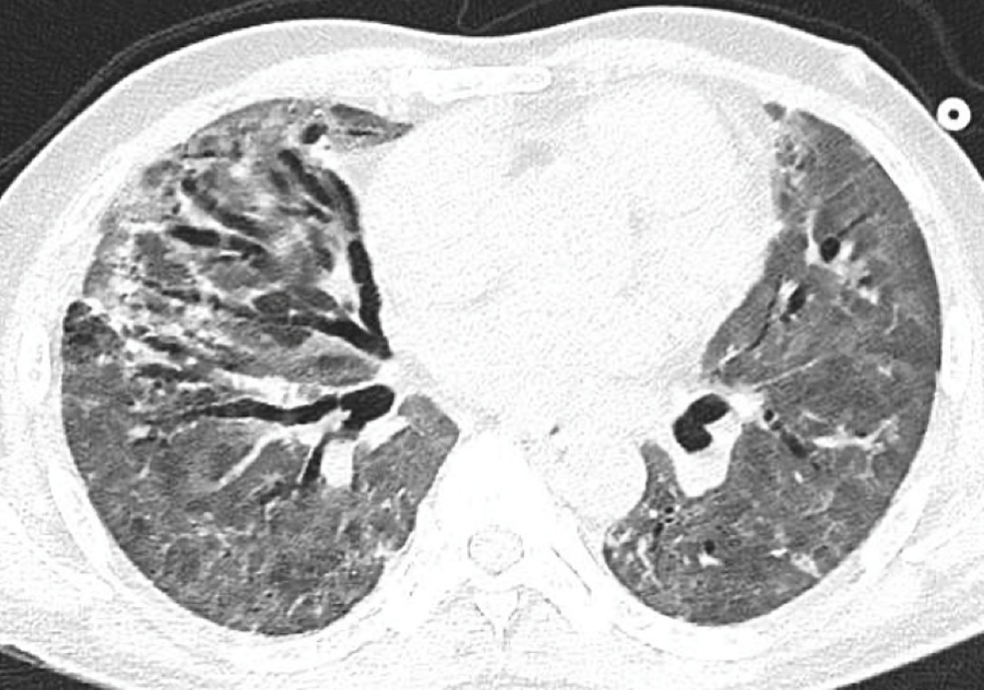
CT chest showing extensive ground-glass opacities in both lung fields with progression of the traction bronchiectasis particularly in the right lung

Over the next few days, the patient was afebrile, however, repeat tracheal aspirate culture was persistently positive for Pseudomonas and Stenotrophomonas maltophilia. Five days later, the patient was extubated but re-intubated again due to respiratory distress and hypoxemia. After multiple failed attempts to wean the patient off mechanical ventilation, the patient was tracheostomized and eventually de-cannulated. The patient was transferred to the medical ward after staying in the critical care unit for a total of 38 days. The patient was on room air when admitted to the medicine ward. However, a few days later, he started to desaturate gradually. Follow-up CXR showed diffuse coarse reticular interstitial changes. COVID-19 rRT-PCR was negative and sepsis workup was unrevealing. A follow-up CT chest showed a slight improvement of the diffuse bilateral ground-glass opacities with re-demonstration of some crazy-paving appearance at both apical segments of the upper lobes, and it also showed progression of the interstitial fibrotic and bronchiectatic changes predominantly in the anterior aspects of both lungs (Figure 4).

The patient was referred to a long-term care hospital for oxygen supplementation, chest physiotherapy, and physical therapy for his critical care myopathy.

## Discussion

Since the initial outbreak of COVID-19, the routine use of CT chest has been useful in detecting early parenchymal lung changes suggestive of COVID-19 infection and in monitoring disease progression, coinfection, or disease stability[2]. Several radiological features were attributed to be classical of COVID-19 infection. A recent systemic review and meta-analysis of 13 studies identified the most encountered CT signs of COVID-19 as being peripheral and bilateral lung involvement with ground-glass opacity (GGO) and consolidation[3].

Bronchiectasis is defined by the presence of permanent and abnormal dilation of the bronchi with CT features of bronchus internal diameter larger than that of its accompanying vessel, lack of bronchial tapering in the periphery of the chest, and visualization of bronchi in the outer 1-2 cm of the lung fields [4]. Gram-negative bacteria are the most frequently identified organisms in the sputum of patients with bronchiectasis. It has been shown to correlate with disease severity, a greater decline in lung function, more frequent exacerbations, and reduced quality of life compared with other bacteria^ ^[5].

Bronchiectasis is not a classical or well-described finding in COVID-19 pneumonia. However, its association has been recently reported. In one retrospective study, bronchiectatic changes were described in one out of 121 COVID-19 patients [6]. Furthermore, a total of four cases of COVID-19 with bronchiectasis were recently reported as well[2,7].

We present a patient with severe COVID-19 pneumonia who developed progressive bronchiectasis within four weeks of symptoms onset. It must be noted that the patient had an evident superadded bacterial infection with Pseudomonas aeruginosa and Stenotrophomonas maltophilia, which required intravenous antibiotics, and a prolonged hospital stay with multiple failed attempts to wean off mechanical ventilation. The complicated course of COVID-19 pneumonia is an anticipated outcome, however, whether the development of bronchiectasis is a contributing factor remains unclear. Worth mentioning is the fact that previously reported cases of COVID-19-induced bronchiectasis showed a paucity of any superadded bacterial infection.

Despite the lack of baseline CT chest of the patient upon presentation, being previously healthy without any preceding hospitalization, a non-smoker, and unrevealing bronchiectasis and tuberculosis (TB) workup supports the suspicion that his rapidly evolving bronchiectasis was induced by COVID-19 pneumonia.

## Conclusions

In conclusion, the COVID-19 pandemic is a public health emergency; nonetheless, its long-term pulmonary complication is not well-studied. The pathophysiology, reversibility, and prognostic implication of COVID-19-associated bronchiectasis require further clinical studies. The main aim of this study is to highlight the rapid progression of bronchiectasis as a sequela of COVID-19 infection, which might impact the prognosis, hence requiring further management and closer follow-up. Follow-up imaging should be considered for patients who had severe COVID-19 and still have persistent pulmonary symptoms. Clinicians should have a high index of clinical suspicion for post-COVID-19 pulmonary sequelae, including pulmonary fibrosis and bronchiectasis.
